# National Insurance and the Poorer Hospitals

**Published:** 1914-07-11

**Authors:** 


### NATIONAL INSURANCE AND THE POORER HOSPITALS.

During the twelve months ended December 31, 1914, 50,000 insured persons have been admitted to in-patient treatment at the voluntary hospitals throughout the country. When it is realised that the cost of treating these insured persons represents a charge on the hospitals of something like £300,000 a year, that not one single penny has been provided by the Chancellor towards the cost of treating these 50,000 in-patients, and that not one single word of acknowledgment has been uttered publicly or privately for the vital service thus rendered by voluntary hospitals to National Insurance, the minds of thoughtful people must be filled with amazed regret. Amazed, because in no circumstances could such an injustice have been done to the thrift instincts of a great people had the scheme of National Insurance been properly thought out and organised before it was embodied in an Act of Parliament. Regret, because the majority of the working-people of these islands are dependent upon the aid rendered to them by the voluntary hospitals throughout the country. The action of the Chancellor of the Exchequer through his insurance scheme in burdening the voluntary hospitals with hundreds of thousands of pounds a year which ought to have been provided for and met, as in Germany, out of Insurance funds, was an exhibition of callousness a statesman would have avoided.

So far as the greater London hospitals are concerned, they have fortunately been in a position up to the present to bear the burden of National Insurance without grievous injury. In the case of the smaller hospitals, many of them—like the Metropolitan Hospital, Kingsland Road, and the Prince of Wales's General Hospital at Tottenham—have been threatened with financial disaster due to the effects of National Insurance upon their revenues. The case of both these hospitals is identical in that each ministers to the medical and surgical requirements of an ever-increasing population amounting to half a million round about Kingsland Road and to upwards of 400,000 at Tottenham. The Metropolitan Hospital supplies a need in one of the poorest districts in London; it has always been a difficult task to raise an adequate income to defray the cost of the good work continuously done there each year, and the effect of recent legislation has been to place the financial position of this and other voluntary hospitals throughout the country in a worse position than they have ever experienced during the previous twenty years. The voluntary hospital managers throughout the country have bravely faced the new problems thus presented, for their hearts are with the suffering and the sick. When will the politicians begin to prove by acts that they too can remember the poor?

Unfortunately, the higher the quality of the hospitals' services and the greater their scientific value, the more costly do they become. It seems a singular proof of an absence of statesmanship that when the functions of our voluntary hospitals were being changed by legislation the politicians responsible provided no money to defray the costs resulting, whilst the voluntary hospital managers were left to fulfil unaided one task made more difficult than ever before—that of the collection of the necessary funds. Sir Frederick Banbury holds that numerous in-patients could contribute something towards the excellent treatment they receive in a voluntary hospital, and that means should be provided to enable each such patient to make these payments. Another step which had been advocated was that power should be given to municipalities and county councils to make grants in aid to the voluntary hospitals, seeing that their continued efficiency was notoriously essential to the maintenance of the earning powers of the working classes especially. Parliament has already given powers, notably in the case of Liverpool, to make such grants-in-aid to existing voluntary hospitals. In view of the large expenditure entailed by the in-patient treatment of insured persons, there could be no proper objection to a course which has been attended with good results in Germany and in many parts of the United States of America. The example set by the Royal Infirmaries at Manchester and Leicester in interesting the Lord Mayor and Corporation and the principal county authorities in the management of these hospitals, might be helpfully followed by hospital managers throughout the United Kingdom.

